# Dataset for quantitative phospho-proteomics analysis of a serial hepatoma cell lines with increasing invasion and metastasis potential

**DOI:** 10.1016/j.dib.2019.104634

**Published:** 2019-10-14

**Authors:** Xiaohua Xing, Hui Yuan, Ying Sun, Kun Ke, Xiuqing Dong, Hui Chen, Xiaolong Liu, Bixing Zhao, Aimin Huang

**Affiliations:** a The United Innovation of Mengchao Hepatobiliary Technology Key Laboratory of Fujian Province, Mengchao Hepatobiliary Hospital of Fujian Medical University, Fuzhou, 350025, People's Republic of China; b The School of Basic Medical Sciences, Fujian Medical University, Fuzhou, 350004, People's Republic of China; c The First Affiliated Hospital of Fujian Medical University, Fuzhou, 350007, People's Republic of China

**Keywords:** Hepatoma, Quantitative phospho-proteomics, FLNA^Ser2152^, ANXA2^Tyr23^, Prognosis

## Abstract

Hepatoma is one of the most common malignant tumor, and most patients have very poor prognosis. Early prediction and intervention of the hepatoma recurrence/metastasis are the most effective way to improve the patients' clinical outcomes. Here, we used isobaric tags for relative and absolute quantitation (iTRAQ) based quantitative phospho-proteomics approach to identify biomarkers associated with hepatoma recurrence/metastasis in hepatoma cell lines with increasing metastasis ability. In total, 75 phosphorylated peptides corresponding to 60 phosphoproteins were significantly dysregulated. Bioinformatics analysis (GO, KEGG and IPA) allowed these data to be organized into distinct categories. These data represent the first in-depth proteomics analysis of a serial hepatoma cell lines with increasing invasion and metastasis potential. The data are related to (Xing et al., 2019).

**Table:** Specifications Table

Subject area	Biology
More specific subject area	Phospho-proteomics on a serial hepatoma cell lines with increasing invasion and metastasis potential
Type of data	Raw, List of identified proteins, phosphopeptides as tables (.xls)
How data was acquired	The data was acquired by Liquid chromatography mass spectrometry in tandem (LC-MS/MS). The samples were separated by an EASY-nLC1000 system (Thermo Fisher Scientific, Bremen, Germany) and detected by a quadrupole-Orbitrap mass spectrometer (Q-Exactive Plus) (Thermo Fisher Scientific, Bremen, Germany).
Data format	Raw, Filtered and analyzed
Experimental factors	A serial hepatoma cell lines with increasing invasion and metastasis potential (Hep3B-a nonmetastatic HCC cell line, HepG2-a lowly metastatic HCC cell line, MHCC97L-a moderately metastatic HCC cell line, MHCC97H-a highly metastatic HCC cell line) were used to comprehensively and systematically investigate the alternations of the proteome and phospho-proteome through iTRAQ-based quantitative phosphoproteomics approach (LC-MS/MS). The samples were labeled with the iTRAQ 8-plex reagent as follows: four groups (Hep3B, HepG2, MHCC97L and MHCC97H) were labeled with 113, 114, 115 and 116 isobaric tag, respectively; and the peptides from the biological repetitions of above 4 groups were labeled with 117, 118, 119 and 121, respectively. Meanwhile, the proteomics dataset was performed with the same as strategy described above and used to normalize the change of phosphopeptide abundance against the change in total protein levels, avoiding fluctuations due to variations in protein amount. For phosphoprotomics, the combined and dried digest was subjected to phosphopeptide enrichment using Magnetic Titanium Dioxide Phosphopeptide Enrichment Kit.
Experimental features	Proteins were extracted form a serial hepatoma cell lines with increasing invasion and metastasis potential, iTRAQ labeled, phosphopeptide enriched and then prepared for liquid chromatography-massspectrometry (LC-MS/MS) analysis.
Data source location	Fuzhou, China, Mengchao Hepatobiliary Hospital of Fujian Medical University
Data accessibility	Repository name: iProX Data identification number: IPX0001763001 Direct URL to data: https://www.iprox.org/
Related research article	Author's name: Xiaohua Xing, Hui Yuan, Ying Sun, Kun Ke, Xiuqing Dong, Hui Chen, Xiaolong Liu, Bixing Zhao, and Aimin Huang Title: ANXA2Tyr23 and FLNASer2152 Phosphorylation Associate with poor prognosis in Hepatic carcinoma revealed by Quantitative hosphoproteomics analysis Journal: Journal of Proteomics https://doi.org/10.1016/j.jprot.2019.03.017

**Table:** 

**Value of the Data** • The first in-depth proteomics and phospho-proteomics analysis of a serial hepatoma cell lines with increasing invasion and metastasis potential. • The first proteome and phospho-proteome profile of a serial hepatoma cell lines with increasing invasion and metastasis potential, which might be useful for further study the mechanisms of hepatoma recurrence and metastasis. • These data could provide a theoretical basis for the follow-up researches on the mechanisms of hepatoma recurrence and metastasis.

## 1 Data

The dataset contains raw sequencing data obtained through the phospho-proteome from a serial hepatoma cell lines with increasing invasion and metastasis potential (Hep3B-a nonmetastatic HCC cell line, HepG2-a lowly metastatic HCC cell line, MHCC97L-a moderately metastatic HCC cell line, MHCC97H-a highly metastatic HCC cell line). The raw data has been available publicly on a data repository (iProX, https://www.iprox.org/) with an accession ID as IPX0001763001. The data are related to [1].

## 2 Experimental design, materials and methods

### 2.1 Experimental design

A serial hepatoma cell lines with increasing invasion and metastasis potential (Hep3B-a nonmetastatic HCC cell line, HepG2-a lowly metastatic HCC cell line, MHCC97L-a moderately metastatic HCC cell line, MHCC97H-a highly metastatic HCC cell line) were used to comprehensively and systematically investigate the alternations of the proteome and phospho-proteome through iTRAQ-based quantitative phosphoproteomics approach (LC-MS/MS). We expect to be able to discover novel potential biomarkers and therapeutic targets associated with invasion and metastasis of hepatoma, and further explain the metastasis/recurrence mechanisms. The samples were labeled with the iTRAQ 8-plex reagent as follows: four groups (Hep3B, HepG2, MHCC97L and MHCC97H) were labeled with 113, 114, 115 and 116 isobaric tag, respectively; and the peptides from the biological repetitions of above 4 groups were labeled with 117, 118, 119 and 121, respectively. Meanwhile, the proteomics dataset was performed with the same as strategy described above and used to normalize the change of phosphopeptide abundance against the change in total protein levels, avoiding fluctuations due to variations in protein amount. For phosphoprotomics, the combined and dried digest was subjected to phosphopeptide enrichment using Magnetic Titanium Dioxide Phosphopeptide Enrichment Kit (Thermo Fisher Scientific, Bremen, Germany).

### 2.2 Materials and methods

Cells were harvested and extracted with lysis buffer (9M Urea, 10 mM Tris-HCl (pH 8.0), 75 mM NaCl, 10 mM IAA, 1 mM NaF, 1 mM β-glycerophosphate, 1 mM Sodium orthovanadate (Na3VO4), 1 mM Sodium pyrophosphate, 1 mM sodium dihydrogen phosphate, 1 mM PMSF, 1% phosphatase inhibitor cocktail 2 (Sigma, St. Louis, MO USA), 1% phosphatase inhibitor cocktail 3 (Sigma)), and 1 tablet of EDTA-free protease inhibitor cocktail (Roche, Basel, Switzerland) for every 10 mL lysis buffer. The cell lysate was sonicated on ice for 1 s followed by a 5 s rest for 8 min. The mixture was centrifuged at 17,000 g for 10 min at 4 °C to remove cell debris. The protein concentration of the supernatant was determined by BCA assay (TransGen Biotech, Beijing, China) following the manufacturer's protocol. After that, the protein mixture was reduced by 10 mM DTT at 55 °C for 30min and alkylated by 50 mM IAA in the darkness at room temperature for 30 min.

The protein digestion and iTRAQ labeling followed our previous reported procedures with modification [2]. Briefly, 100μg proteins were digested *via* the FASP protocol with spin ultrafiltration units with molecular weight cut off 10,000 Da. Then, proteins were digested with trypsin, and the peptide mixture was further labeled using the iTRAQ reagent kit (AB SCIEX, USA). Peptides were labeled with the iTRAQ 8-plex reagent respectively. Equal amount of 8 labeled samples were combined and cleaned up by Sep-Pak Vac C_18_ cartridges (Waters) and then dried in a vacuum centrifuge for further usage.

For phosphoprotomics, the combined and dried digest was subjected to phosphopeptide enrichment using Magnetic Titanium Dioxide Phosphopeptide Enrichment Kit (Thermo Fisher Scientific, Bremen, Germany). Briefly, TiO_2_ magnetic beads were firstly washed by binding buffer three times. Then the peptide mixtures were incubated with magnetic beads in loading buffer (80% ACN, 2% FA). And then, the magnetic beads were washed in turn by binding buffer and washing buffer. Finally, the phosphopeptides were eluted by elution buffer and dried in a vacuum centrifuge for further usage.

The LC-MS/MS analysis follows our previously reported protocol with certain modification [2]. Briefly, the phosphopeptides were respectively re-suspended with 5 μL solvent A (0.1% formic acid in water), and separated by an EASY-nLC1000 system (Thermo Fisher Scientific, Bremen, Germany) and analyzed by a quadrupole-Orbitrap mass spectrometer (Q-Exactive Plus) (Thermo Fisher Scientific, Bremen, Germany) equipped with an online nano-electrospray ion source. 4 μL peptides was loaded onto the trap column (Thermo Scientific Acclaim PepMap C18, 100 μm × 2 cm) with a flow of 10 μL/min, and subsequently separated on the analytical column (Acclaim PepMap C18, 75 μm × 150 cm) with a gradient of 2–8% solvent B for 15min, 8–15% solvent B for 30 min, 15–32% solvent B for 25 min, 32–80% solvent B for 1 min followed by isocratic conditions at 80% solvent B for 5 min with a flow rate of 300 nl/min at 40 °C.

The Q-Exactive Plus mass spectrometer was operated in the data-dependent mode to switch automatically between MS and MS/MS acquisition. The electrospray voltage of 2.1 kV at the inlet of the mass spectrometer was used. Survey full-scan MS spectra (*m*/*z* 350–1200) was acquired with a mass resolution of 70 K, followed by 15 sequential high energy collisional dissociation (HCD) MS/MS scans with a resolution of 17.5 K. In all cases, one microscan was recorded using dynamic exclusion of 30 seconds.

Immunohistochemistry was performed on hepatocellular carcinoma tissue microarray as previously described [3]. Briefly, following pretreatment at pH 6 and peroxidase blocking, the slides were respectively incubated with the primary antibody (*anti*-p-FLNA, 1/200 dilution and *anti*-p-ANXA2, 1/200 dilution) for 30 min. Then, the slides were washed, treated with the envision FLEX/HRP system for 20 min and revealed with the envision FLEX-DAB chromogen (Dako) and with Mayer's Hematoxylin (Lille's Modification) Histological Staining Reagents (Dako 53309) for 3 minutes and distilled by water for 5 minutes.

### 2.3 Data analysis

MS data acquiring was processed using Proteome Discoverer (Thermo Fisher Scientific, Bremen, Germany; version 1.4) against the uniprot human_database (released at 2014-04-10, 20,264 entries). The complete list of identified proteins and phosphopeptides in our study was shown in Tables 1 and 2 in Supplementary data.

To identify phosphopeptides whose phosphorylation level was significantly altered with increasing invasion and metastasis potential, we set a threshold of fold change >1.5 or <0.67 (log_2_ ratio lower than −0.58 or higher than 0.58). In order to screen abnormal phosphorylation contribute to the invasion and metastasis of hepatoma cells, we focused on the most reliable quantitative data, i.e. phosphoproteins and phosphopeptides that the abundance was steady increased or decreased from the lowest metastatic cell line to the highest metastatic cell line (from Hep3B to MHCC97H). Despite this very stringent criterion, we found that 75 phosphopeptides corresponding to 60 phosphoproteins (Table 3 in Supplementary data) were steadily changing their abundance according with the metastatic potential, revealing the important phosphorylation events associated with the metastasis ability of cell lines.

### 2.4 Bioinformatic analysis

**Fig. 1:**
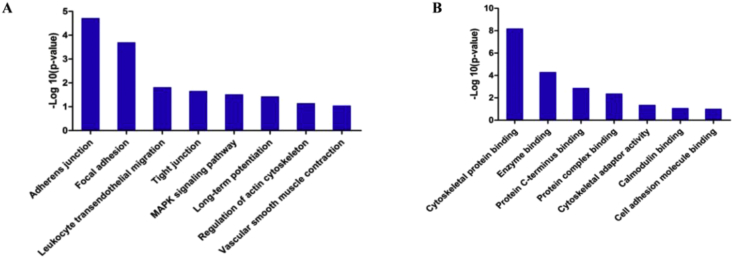
The involved biological processes (A) and molecular functions (B) of the dysregulated phosphoproteins by GO analysis.

The Gene Ontology (GO) annotation and pathway enrichment analysis of all the identified proteins and differentially expressed proteins were implemented using the online tool DAVID (http://david.abcc.ncifcrf.gov/). The GO analysis about biological processes and molecular functions of the dysregulated phosphoproteins were displayed in Fig. 1. The GO annotations were ranked in term of the enrichment of the differentially expressed proteins.

**Fig. 2:**
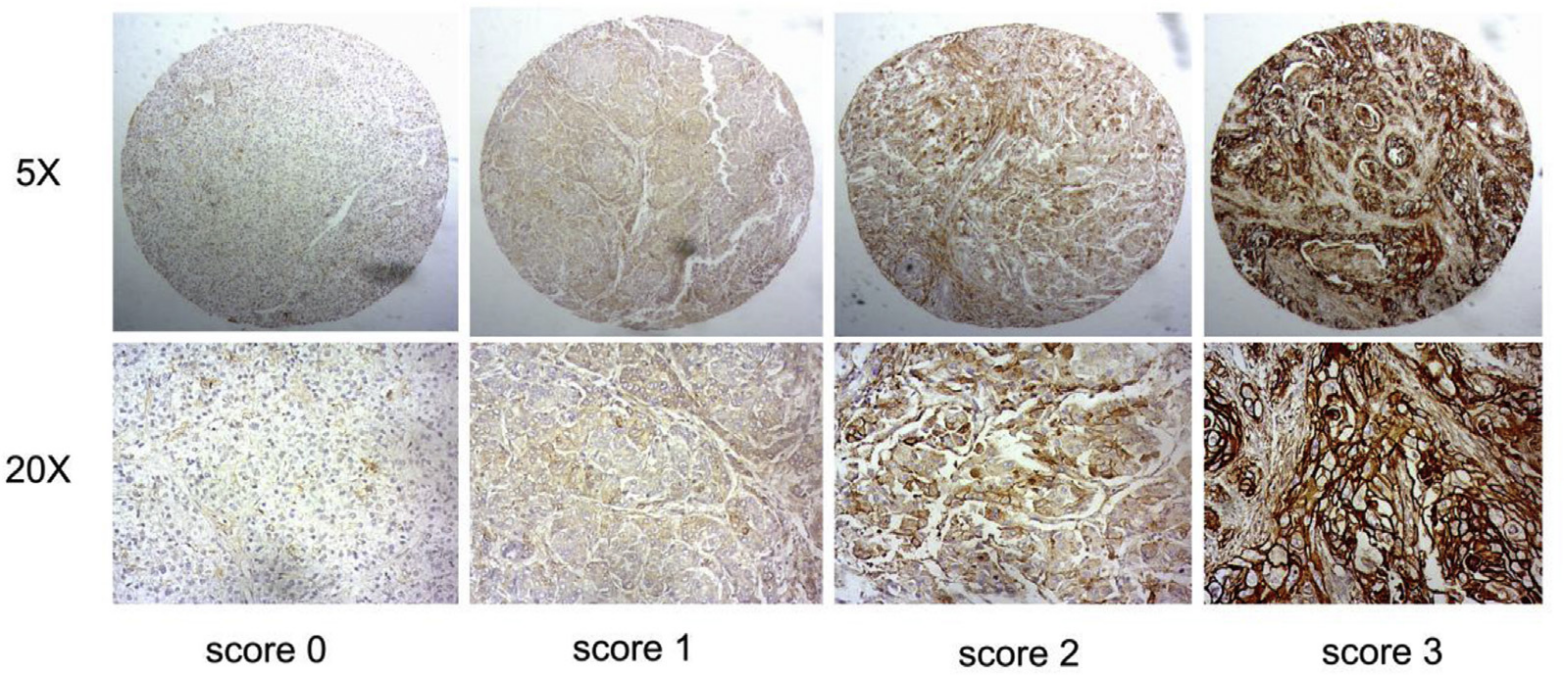
The presentative images of ANXA2 immunostaining in tumor tissues and its corresponding adjacent non-tumor tissues of different groups. Scale bar, 50μm, *p < 0.05.

**Fig. 3:**
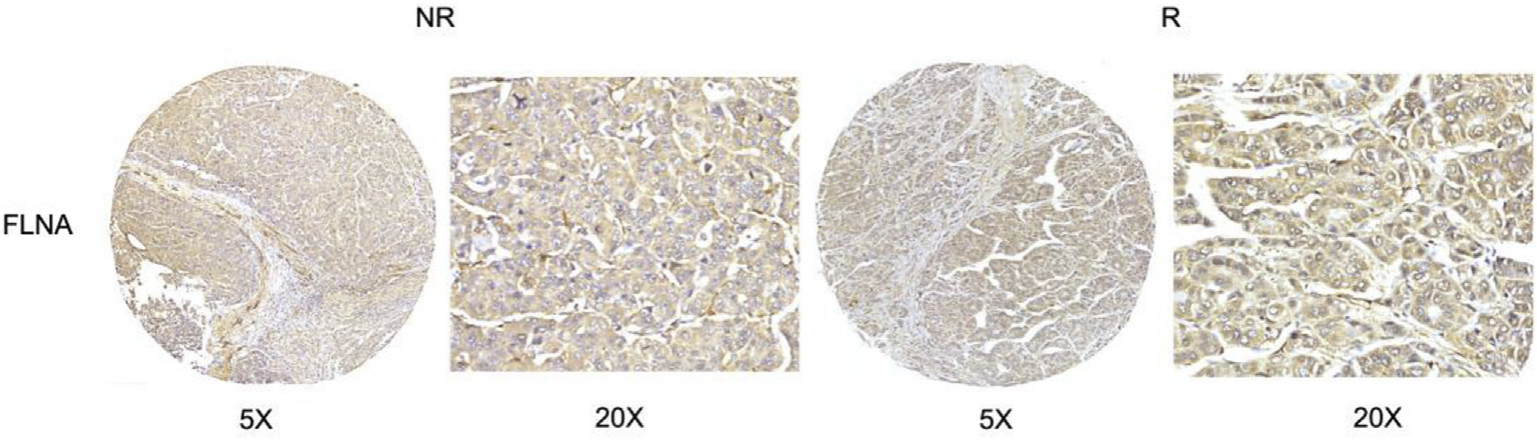
The levels of FLNA validated by IHC are not changed in the recurrent hepatoma patients comparing with nonrecurrent hepatoma patients.

To further confirm the correlation between these two phosphorylation events and recurrence and metastasis of hepatoma in patients, alternation pattern of these two phosphoproteins at phosphorylation level was performed in a cohort of 80 HCC patients using tissue microarray. The IHC results were independently assessed by two pathologists double-blindly. All tissues were manually scored as 0 (negative), 1 (weak), 2 (strong) or 3 (very strong). Each case was considered to be low expression if the final score was 0–1, while high expression if the final score was 2–3 (Fig. 2). The representative images of ANXA2 immunostaining in hepatoma tissues were shown in Fig. 3.
